# The effect of music on nonstress test and pregnant women's satisfaction: a randomized controlled trial

**DOI:** 10.1590/1806-9282.20240797

**Published:** 2024-12-02

**Authors:** Yasemin Sökmen, Resmiye Kaya Odabaş, Bahadır Yazıcıoğlu

**Affiliations:** 1 Ondokuz Mayıs University, Faculty of Health Sciences, Department of Midwifery – Samsun, Turkey.; 2 Kocaeli University, Faculty of Health Sciences, Department of Midwifery – Kocaeli, Turkey.; 3 Samsun Provincial Health Directorate, Samsun, Turkey.

**Keywords:** Cardiotocography, Fetal heart, Music, Satisfaction, Pregnancy

## Abstract

**OBJECTIVE:**

The aim of this research was to determine the effect of music on nonstress testing and pregnant women's satisfaction.

**METHODS:**

The research, which was designed as a randomized controlled experimental study, was conducted between September 2021 and September 2022 at a training and research university hospital in the north of Turkey. The population of the research consisted of pregnant women who were requested to take a nonstress test, and the sample consisted of 111 pregnant women (music group: 56 and control group: 55). The pregnant women in the music group listened to classical music during the nonstress test, while no intervention was applied to those in the control group. Data were collected with a Descriptive Information Form, a Nonstress Test Form, and the Visual Analog Scale to evaluate satisfaction. Mann-Whitney U and chi-square test statistics were used for the statistical analysis of the data.

**RESULTS:**

The mean basal fetal heart rate and count of fetal movements, the presence of acceleration, and the nonstress test results of the pregnant women in the music group were statistically significantly higher than those of the control group (p<0.05). Besides, a statistically significant difference was found between the groups in terms of mean satisfaction scores (p<0.05).

**CONCLUSION:**

Listening to music during nonstress tests increases pregnant women's reactive nonstress test rates and satisfaction.

## INTRODUCTION

Fetal death is an important public health problem, accounting for approximately half of perinatal deaths. The global stillbirth rate in 2021 was 13.9 per 1,000 births^1^. The goal of the Every Newborn Action Plan is to reduce the national stillbirth rate of all countries to below 12 per 1,000 births by 2030^2^. Therefore, it is important to evaluate fetal health to identify fetuses at risk of intrauterine death. Ultrasound, Doppler ultrasound, maternal evaluation of fetal movements, biophysical profile, nonstress test (NST), and contraction stress test are utilized to assess fetal health. A nonstress test is the evaluation of changes in fetal heart rate during spontaneous or stimulated fetal movements^3^.

Music is an energy consisting of regular sounds and vibrations^4^. In a systematic review of the effect of music on pregnant women's health, it was reported that playing music to pregnant women had a positive effect on pain, stress, anxiety, depression, sleep, satisfaction, maternal–fetal attachment, and NST parameters^5^. This is because music increases the secretions of natural serotonin and acetylcholine, reduces the release of glucocorticoids such as cortisol that maintains heart rate and blood pressure, and increases blood flow to vital organs^5^.

It has been reported that taking a nonstress test is time-consuming, boring, and a source of anxiety for the mother, the procedure time is prolonged or repeated due to fetal sleep and the rate of false nonreactive results is high, and therefore, invasive interventions such as cesarean sections are increasing^3^. Some studies on the effect of music during a nonstress test have shown that while music increases the basal fetal heart rate, variability, number of fetal movements, number of accelerations, and reactive NST results, it reduces pregnant woman's anxiety, the count of decelerations, and the duration of NST^3,6,7^.

One of the basic principles of quality in health services is the satisfaction of the service recipient and provider^8^. Patient satisfaction is an indicator of how satisfied patients are with the healthcare service they receive from a healthcare professional^9^. In addition, women's comfort, satisfaction, and cooperation during NST may affect the NST results^10^. Considering the relaxing and positive effects of music, it is thought that it will increase satisfaction by relaxing women during NST. While there is limited research in the literature on the effect of music on NST and pregnant women's anxiety^3,6^, there are no studies on the effect of music on pregnant women's satisfaction during NST. Therefore, it was thought that this study would contribute to the literature. It is thought that examining the effect of music from different genres and cultures on NST will contribute to the literature by providing new data about non-pharmacological methods that can be applied during fetal health assessment. The aim of this study was to determine the effect of music on NSTs and pregnant women's satisfaction.

## METHODS

### Type of the study

This is a randomized controlled experimental study.

### Setting of the study

The research was conducted in the NST room of a training and research hospital in Samsun province in northern Turkey between September 2021 and 2022.

### Population and sample of the study

The sample size of the study was calculated on the G*Power 3.1.9.2 software, based on data from a study by Kafali et al^11^. The mean and standard deviation values of the basal fetal heart rate in the aforementioned study were used, and the effect size was calculated as 0.69. The minimum number of individuals that was needed in the sample of this research was calculated as 92 pregnant women (including 46 in each group) on the G*Power 3.1.9.2 software, based on an effect size of 0.69, an alpha value of 0.05, and a power value of 0.95. The number of samples was increased by 20% to boost the analysis power of the research, and therefore a total of 112 pregnant women (music group: 56 and control group: 56) were included in the study. The study started with 56 pregnant women in the music group and 56 in the control group, but one pregnant woman in the control group who had a labor contraction was excluded from the study. Thus, the study was completed with a total of 111 participants, including 56 in the music group and 55 in the control group.

### Inclusion and exclusion criteria

The study included pregnant women who were between the ages of 18 and 35, were in their ≥34th gestational week, and had a singleton and a live pregnancy. Those who had uterine contractions as a result of NST, had a chronic disease (such as diabetes), had a pregnancy complication (such as preeclampsia), had a problem in the fetus (such as intrauterine growth retardation), and used tobacco or alcohol were excluded.

### Data collection tools

The data of the study were collected using a Descriptive Information Form and a Nonstress Test Form created by the researchers, and the Visual Analog Scale (VAS) was used to evaluate satisfaction.

Descriptive Information Form: This form was developed by the researchers in line with the literature^3,6^. It consists of a total of 12 questions about pregnant women's sociodemographic and obstetric characteristics.

Nonstress Test Form: This form was developed by the researchers in line with the literature^3,6,7^. It consists of seven questions about NST (such as fetal heart rate).

The VAS for the Assessment of Satisfaction: It is rated by individuals by putting marks on a 10-cm horizontal line, with one end reading "0" and the other "10," where "0=not at all satisfied" and "10=very satisfied." The pregnant women in the study were told to mark the point between the two extremes that matched their satisfaction level. The distance between the beginning of "I am not satisfied at all" and the point marked by the pregnant woman was measured and recorded in centimeters.

### Preliminary application

A preliminary application was carried out with five pregnant women to evaluate the comprehensibility and applicability of the data collection forms. Necessary modifications and improvements were made based on the observations, and the pregnant women included in the preliminary application were not included in the study.

### Data collection

Before the nonstress test was initiated, participants were interviewed face-to-face, and their sociodemographic and obstetric characteristics were recorded on the Descriptive Information Form. After that, according to the findings of the Leopold maneuvers, the probes of the NST device were placed, and the pregnant women were given a left-side lying position. The pregnant women in the intervention group listened to music during NST, and the control group participants took the NST with routine care. The Descriptive Information Form was filled out face-to-face, the VAS was marked by the pregnant woman, and the Nonstress Test Form was filled out by the first researcher.

The group in which the participants would be placed was determined from the website "www.randomizer.org" and the researchers were kept blind during the assignment to the groups. The VAS for the Assessment of Satisfaction was collected through women's self-report, and the researchers were blinded to the scale results. Blinding was ensured by the analysis of the data by the second researcher who was not involved in the randomization and data collection stages.

### The intervention

The music group: The selection of music to be played to pregnant women was determined based on previous studies on the subject^3^. During the 20-min NST procedure, the pregnant woman listened to some of Beethoven's pieces, namely Für Elise, Moonlight Sonata, and Ode to Joy on a Powerway brand MP3 player.

The control group: After the nonstress test was initiated, no treatment was performed on the pregnant women in this group; only the routine follow-up was performed.

### Data analysis

The study data were evaluated on the SPSS software package. Descriptive statistics were presented using mean, standard deviation, median, minimum, maximum values, and 95% confidence interval for continuous variables and frequency and percentage values for categorical variables. Categorical data were compared using the chi-square test. Continuous data were first evaluated with the Kolmogorov-Smirnov test for normality, and the Mann-Whitney U test was used for the analysis of continuous data. The results were evaluated based on a confidence interval of 95%, and p<0.05 showed statistical significance.

### Ethical aspects of the study

This study was conducted in accordance with the principles of the Declaration of Helsinki. The approval of the Ondokuz Mayıs University Clinical Research Ethics Committee (Protocol number: OMÜ KAEK 2021/280; Date: 11.06.2021) was obtained to conduct the research and collect data. Additionally, the study was registered with Clinical Trials (ID number: NCT06137872). The pregnant women included in the study were informed about the purpose of the research and the benefits to be gained, and their verbal and written consent was obtained.

## RESULTS

The groups were found to be similar in terms of sociodemographic and obstetric characteristics (p>0.05) (Table 1). This was important as it showed the groups were homogeneous and comparable.

**Table 1:** Comparison of sociodemographic and obstetric characteristics of pregnant women in the music and control groups.

Characteristics/Groups		Music (n=56)	Control (n=55)	Test	p-value
		n (%)	n (%)		
Age (year)					
	Mean±SD*	26.96±3.56	27.04±3.63	1413.0	0.381^1^
	Median/min–max	27/20–33	26/20–34		
	95%CI**	26.04–28.02	26.08–27.98		
Level of education					
	Elementary/middle school	20 (35.7)	17 (30.9)	2.268	0.519^2^
	High school	26 (46.4)	28 (50.9)		
	University and above	10 (17.9)	10 (18.2)		
Employment status					
	Yes	9 (16.1)	13 (23.6)	0.580	0.446^2^
	No	47 (83.9)	42 (76.4)		
Weeks of gestation					
	Mean±SD*	36.45±1.58	36.22±1.59	1395.5	0.385^1^
	Median/min–max	36/34–40	36/34–40		
	95%CI**	36.06–36.90	35.82–36.67		
Number of pregnancy					
	Mean±SD*	2.51±1.32	2.33±1.29	1364.0	0.207^1^
	Median/min–max	2/1–5	2/1–5		
	95%CI**	2.16–2.86	1.84–2.49		
Status of curettage					
	Yes	53 (94.6)	51 (92.7)	–	–
	No	3 (5.4)	4 (7.3)		
Status of miscarriage					
	Yes	49 (87.5)	47 (85.5)	–	–
	No	7 (12.5)	8 (14.5)		

* Mean±standard deviation;

** confidence interval;

1 Mann-Whitney U test;

2 Chi-square test statistics. SD: standard deviation; CI: confidence interval.

In our study, pregnant women's mean basal fetal heart rate per minute was 124.11±7.25 in the music group and 122.50±5.96 in the control group. A statistically significant difference was found between the mean basal fetal heart rate of the groups (p=0.011). The variability of 94.6% of the pregnant women in the music group and 87.3% of those in the control group were found to be normal (6–25 beats). The mean count of fetal movements was determined as 15.89±7.16 in the music group and 11.38±7.42 in the control group. A statistically significant difference was found between the mean number of fetal movements between the groups (p<0.001). Acceleration was detected in the NST evaluation of 89.3% of the music group and 65.5% of the control group, and a statistically significant difference was detected between the groups in terms of the presence of acceleration (p=0.003). In our study, the NST results of 89.2% of the pregnant women in the music group and 63.6% of the pregnant women in the control group were determined to be reactive, and a statistically significant difference was detected between the NST results of these two groups (p=0.002) (Table 2).

**Table 2:** Comparison of nonstress test results of pregnant women in the music and control groups.

Characteristics/Groups		Music (n=56)	Control (n=55)	Test	p-value
		n (%)	n (%)		
Basal fetal heart rate (beats/min)					
	Mean±SD*	124.11±7.25	122.50±5.96	1154.0	**0.011** 1
	Median/min–max	120/110–140	120/110–140		
	95%CI**	122.74–126.40	120.10–122.37		
Variability					
	Normal (6–25 beats)	53 (94.6)	48 (87.3)	1.839	0.175^2^
	Decreased (3–5 beats)	3 (5.4)	7 (12.7)		
Number of fetal movements					
	1	0 (0.0)	2 (3.6)	381.5	**0.001** 1
	≥2	56 (100.0)	53 (96.4)		
	Mean±SD*	15.89±7.16	11.38±7.42		
	Median/min–max	15/6–36	10/1–36		
	95%CI**	13.96–17.72	5.95–8.67		
The presence of acceleration					
	Yes	50 (89.3)	36 (65.5)	9.031	**0.001** 2
	No	6 (10.7)	19 (34.5)		
The presence of deceleration					
	Yes	0 (0.0)	3 (5.5)	3.139	0.076^2^
	No	56 (100.0)	52 (94.5)		
Deceleration type (n=0;3)					
	Early	0 (50.0)	2 (66.7)	–	–
	Late	0 (50.0)	1 (33.3)		
Nonstress test result					
	Reactive NST	50 (89.2)	35 (63.6)	9.194	**0.002** 2
	Nonreactive NST	3 (5.4)	15 (27.3)		
	Suspected NST	3 (5.4)	5 (9.1)		

* Mean±standard deviation;

** confidence interval;

1 Mann-Whitney U test;

2 Chi-square test statistics. SD: standard deviation; CI: confidence interval; NST: nonstress test. Bold indicates p<0.05.

The mean satisfaction score of the pregnant women in the music and control groups was 7.49±1.14 and 6.59±1.26, respectively, and a statistically significant difference was detected between the groups in terms of the mean satisfaction score (p<0.001) (Table 3).

**Table 3:** Comparison of Visual Analog Scale for the Assessment of Satisfaction of pregnant women in the music and control groups.

Characteristics/Groups		Music (n=56)	Control (n=55)	Test	p-value
		n (%)	n (%)		
The VAS for the Assessment of Satisfaction					
	Mean±SD*	7.49±1.14	6.59±1.26	342.0	**0.001** 1
	Median/min–max	7/5–10	7/5–10		
	95%CI**	7.34–7.95	5.65–6.08		

* Mean±standard deviation;

** confidence interval;

1 Mann-Whitney U test. SD: standard deviation; CI: confidence interval; VAS: Visual Analog Scale. Bold indicates p<0.05.

## DISCUSSION

In this study, which was conducted to evaluate the effect of listening to music during the nonstress test procedure on NST and pregnant women's satisfaction, the NST result of the majority of the pregnant women in the music group was reactive, while this rate was lower in the control group. Similar to our research findings, some studies in the literature have shown that listening to music during NST increases the reactive NST results^3,6,12-17^. Contrary to our research findings, Soylu et al.^17^ found no difference between the groups in terms of NST results. We think that revealing the controversial results in the literature through meta-analysis studies will end the confusion in the literature.

Pregnant women's satisfaction level during healthcare services increases both communication and the quality of care^10,18-20^. In our study, the mean satisfaction score of the music group was found to be higher than that of the control group. Similar to our research findings, Yılmaz Sezer et al.^20^ reported that the mean scores of the pregnant women in the experimental group on the satisfaction scale were higher than the scores of the control group. It is thought that individuals who are satisfied with the service they receive will come to the health institution with more positive feelings in their next presentation and that this will indirectly be reflected in the NST result. Therefore, music application, which is easy to apply, low-cost, and noninvasive, can be recommended to be applied by midwives during NST procedures.

## CONCLUSION

In our study, it was concluded that listening to music was effective in increasing the reactive NST results of pregnant women and their satisfaction. It is recommended that health professionals use music, which is noninvasive, natural, safe, easy-to-use, cost-free, and side-effect-free, during NST procedures, midwives should be given in-service training on the positive effect of music on NST.

## ETHICS APPROVAL STATEMENT

Consent of Ondokuz Mayıs University Clinical Research Ethics Committee on 11.06.2021 with OMÜ KAEK 2021/280 number with decree number was taken. The study was registered with Clinical Trials (ID number: NCT06137872).
